# Highly Pathogenic Avian Influenza Virus, Midwestern United States

**DOI:** 10.3201/eid2201.151053

**Published:** 2016-01

**Authors:** Chau M. Bui, Lauren Gardner, C. Raina MacIntyre

**Affiliations:** University of New South Wales School of Public Health and Community Medicine, Sydney, New South Wales, Australia (C. Bui, C.R. MacIntyre);; University of New South Wales School of Civil and Environmental Engineering, Sydney (L. Gardner)

**Keywords:** epidemiology, influenza in birds, North America, viruses, influenza virus, highly pathogenic influenza virus, HPAI, respiratory infections

**To the Editor:** Novel highly pathogenic avian influenza (HPAI) viruses of subtypes H5N2, H5N8, and H5N1 have recently caused numerous outbreaks in commercial poultry farms in the United States and Canada (*1*). Risk for zoonotic transmission is low; humans are affected primarily from the extensive economic repercussions of suspending poultry-farming activities (*1*).

Large-scale research is under way, including case-control studies of infections on poultry farms and modeling studies to investigate the spread of virus in waterfowl (*1*,*2*). The US Department of Agriculture has published a report that summarizes various biosecurity measures of affected farms, results of airborne pathogen testing, and geospatial analyses correlating wind speed and direction to outbreaks (*1*). These studies found insufficient evidence to support any particular modes of virus spread and suggest that farms are contaminated from infected migrating waterfowl and/or unauthorized movements (e.g., of vehicles, equipment, persons, or animals) between farms and that unusually high wind speeds are the likely mechanism of spread (*1*). The spread from farm to farm, but not from barn to barn within a single farm (*3*), further adds to the puzzle of how infection has been transmitted.

To better understand the outbreak behavior, we used publicly available sources (*4*–*6*) to create maps of outbreaks of HPAI virus, subtype H5, infections in relation to poultry distribution and wild bird migratory patterns (Figure; Technical Appendix Figures 1, 2; Video). From November 30, 2014, through June 17, 2015, a total of 280 outbreaks caused by HPAI virus subtype H5 in Canada and the United States were reported to the World Organisation for Animal Health (*4*). Most outbreaks occurred during April (n = 116) in commercial turkey farms (n = 154) and were caused by HPAI virus subtype H5N2 (n = 256) (Technical Appendix Figure 3). Related reassortant HPAI subtypes H5N8 and H5N1 were also found among infected poultry; however, these appeared infrequently. Subtype H5N1 appeared in 4 of 21 outbreaks in backyard and commercial farms and was found in 1 of 3 infections in a backyard farm. Backyard farms generally contain flocks for local consumption and implement fewer biosecurity measures (*4*).

**Figure:**
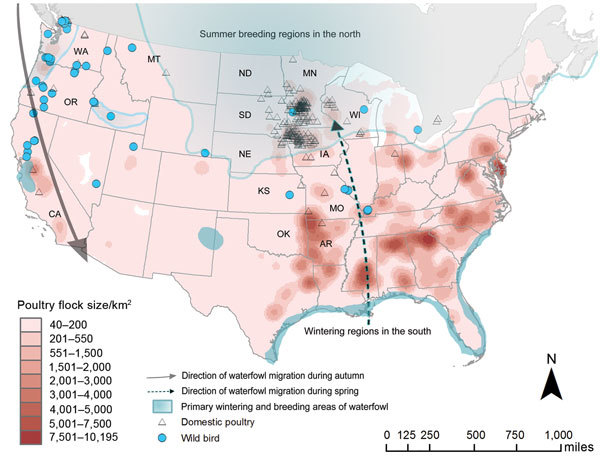
Distribution of outbreaks caused by highly pathogenic avian influenza (HPAI) virus, subtype H5, in domestic poultry compared with domestic poultry flock density and direction of wild waterfowl migration. Triangles represent outbreaks caused by HPAI virus, subtype H5, in domestic poultry; blue circles represent HPAI virus, subtype H5 outbreaks in wild birds. Blue shading indicates migratory waterfowl wintering and breeding regions, and arrows represent general direction of seasonal movements. Pink shading indicates density of domestic poultry holdings, with darker shades representing areas where flock densities are higher.

**Video:**
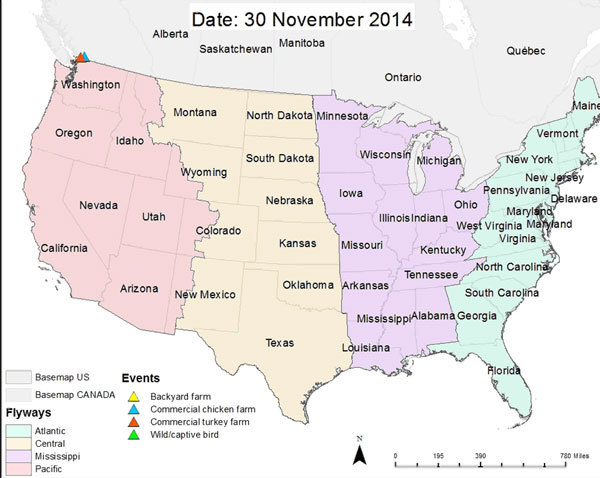
Time-series map showing the cumulative daily geographic distribution of highly pathogenic avian influenza (HPAI) virus, subtype H5, events in North America, from November 30, 2014, to May 18, 2015. Events are symbolized by triangles. The different colors show which bird or farm type was affected: green, wild bird; yellow, backyard farm; red, commercial turkey farm; blue, commercial chicken farm; pink, Pacific flyway; brown, central flyway; purple, Mississippi flyway; green, Atlantic flyway.

Initial outbreaks on poultry farms that began in November 2014, near the British Columbia–Washington State border, have been associated with timing of waterfowl migration and reported infection in migratory waterfowl (*7*,*8*). Subsequent surveillance of avian influenza virus in wild birds in the Pacific flyway has also shown sporadic infections caused by HPAI virus subtype H5, primarily in waterfowl species of the family Anseriformes (*4*) (Technical Appendix Table 1).

In late February 2015, however, HPAI virus subtype H5, emerged in US midwestern states, leading to a substantial number of outbreaks in commercial poultry farms in the region. The spread from west to east does not correlate with the direction of typical waterfowl migration, in which movement occurs from south to north. Unlike the earlier outbreaks in poultry in Canada, in the outbreaks in midwestern states, corresponding high numbers of virus were not detected in samples of wild birds in surrounding regions (despite increased surveillance). Of 3,300 samples tested, 1 sample tested positive for HPAI virus subtype H5 (*4*,*9*). In addition, most poultry farms were affected in April, and migratory waterfowl typically appear in Minnesota in March and April (Technical Appendix Figure 1). This February introduction of virus to Minnesota may be explained by an earlier-than-usual spring (*10*).

Minnesota and Iowa lie within regions where migrating waterfowl spend their breeding season, and waterfowl densities on commercial poultry farms are particularly high (Technical Appendix Figure 2). In southern parts of the United States, where poultry density is also high, isolated outbreaks of HPAI have occurred in poultry, although the introduction of virus into these regions did not result in a surge of outbreaks. The timing of waterfowl migration enables the mixing of highly dense populations of wild waterfowl and poultry, which likely plays a key role in spreading virus onto farms.

Of particular note, outbreaks in poultry were densely concentrated within Minnesota and Iowa in a spatial pattern inconsistent with the much more geographically dispersed spread of infection in wild birds. The magnitude and clustered distribution of poultry outbreaks are suggestive of local spread, rather than multiple introductions from passing migratory waterfowl. Genetic analyses have similarly shown evidence for concurrent multiple introductions as well as common source exposures, and surveys of affected farms have shown that local spread could be facilitated by the sharing of equipment by multiple farms or through animals entering barns (*1*).

The combination of high poultry densities and timing of waterfowl migration have likely predisposed Minnesota and Iowa to outbreaks of avian influenza among poultry flocks. However, consistent with US Department of Agriculture findings, local factors have likely also contributed to the large number of outbreaks in these states. We suggest that network modeling analyses would be valuable in exploring how virus may spread from farm to farm.

Technical Appendix The Technical Appendix describes materials and methods used in the study of outbreaks caused by highly pathogenic influenza (HPAI) virus, subtype H5, in North America
