# Supplementary material for: Highly Pathogenic Avian Influenza Virus, Midwestern United States
Source: Emerg Infect Dis. 2016 Jan;22(1):138–9. doi: 10.3201/eid2201.151053 (PMC4696700; doi:10.3201/eid2201.151053)
Supplement: Technical Appendix — The Technical Appendix describes materials and methods used in the study of outbreaks caused by highly pathogenic influenza (HPAI) virus, subtype H5, in North America [file 15-1053-Techapp-s1.pdf]

Article DOI: http://dx.doi.org/10.3201/eid2201.151053

Highly Pathogenic Avian Influenza Virus, Midwestern United States

Technical Appendix

Materials

Coordinate data on confirmed outbreaks caused by highly pathogenic avian influenza (HPAI), subtype H5, were available from the World Organisation for Animal Health (OIE) (*1*). Poultry farm locations and flock sizes were obtained from a modeled dataset designed for use in research (*2*). This dataset contains estimated coordinate locations of poultry farms, along with estimates of farm-holding size and reflect US census data. Estimated distributions of wild bird populations were derived from bird-banding data supplied by the US Geological Survey, Patuxent Wildlife Research Centre and Bird Banding Laboratory Canadian Wildlife Service, National Wildlife Research Centre (available on request at http://www.pwrc.usgs.gov/. A list of all wild bird species reporting HPAI H5 viruses (Technical Appendix Table 1) was compiled using information from http://www.aphis.usda.gov/. Generalized distributions of waterfowl wintering and breeding regions were obtained from the revised edition of Waterfowl of North America (*3*). Data on flyways in North America were available at http://www.ducks.org/.

Methods

ArcMap software (Version 10.2) was used to create maps (Figures 1; Technical Appendix Figures 1, 2) and a time-series video of outbreaks (Video). Base maps of the United States and Canada were obtained from the GADM database of Global Administrative Areas (http://www.gadm.org/). The North America Lambert Conformal Conic projection **(**www.esri.com**)** was used for all layers. Adobe Photoshop, version CS5 (https://www.adobe.com), was used for graphics editing.

Disease event data from OIE (*1*) were transcribed into a line list on Microsoft Excel (Microsoft Corp., Redmond, WA, USA). OIE reports from the United States and Canada contain the following: start date of disease event, sample collection location (country, state, county, and occasionally a more specific location name), coordinate data, the species of bird affected, and if a farm was reported, the farm type affected (commercial or backyard). Coordinate data of disease events were used to plot the location of HPAI H5 outbreaks. Epidemic curves were created by using Microsoft Excel (Technical Appendix Figure 3).

Coordinate data representing synthetic poultry farm locations were plotted for each of the 3 domestic poultry groups: chicken farms (broilers, layers, pullets), turkey farms, and other poultry farms (duck, geese, other) (Technical Appendix Figure 2, panels A, C, E). We additionally plotted large flock farms that house >10,000 poultry on separate maps for each of the 3 poultry groups (Technical Appendix Figure 2, panels B, D, F). Kernel density transformations were also performed, using all 3 poultry groups together (Figure 1).

Coordinate data from the bird-banding dataset was used to create maps displaying wild bird encounter locations for each month of the study period (Technical Appendix Figure 1, panels A–F). Encounter locations refer to locations where banded wild birds have been found (dead, hunted, or recaptured). Further information can be found at http://www.pwrc.usgs.gov/BBL/MANUAL/Howobt.cfm. Data were limited to recent bird-banding data (January 2013 onwards) and restricted to the 11 Anseriformes species (waterfowl) that have had reported HPAI H5 viruses as species from the Anseriformes and Charadriiformes families (shorebirds), which are considered to be the natural reservoirs of avian influenza viruses. For each month, the kernel density of bird encounters (per km^2) were calculated and displayed with the plotted disease incidence for that particular month. Generalized waterfowl seasonal distributions from Waterfowl of North America, revised edition (*3*) and direction of seasonal movement were digitized by using Adobe Photoshop (version CS5) (see Figure 1).

References

1. World Organisation for Animal Health (OIE). OIE reports. 2015. [cited 2015 Jul]. http://www.oie.int

2. Bruhn MC, Munoz B, Cajka J, Smith G, Curry RJ, Wagener DK, et al. Synthesized population databases: a geospatial database of US poultry farms. RTI Press publication no. MR-0023-1201.

Research Triangle Park (NC): RTI Press; 2012 [cited 2015 Jul]. http://www.rti.org/pubs/mr-0023-1201-bruhn.pdf http://dx.doi.org/10.3768/rtipress.2012.mr.0023.1201

3. Johnsgard PA. Waterfowl of North America, rev. ed. Lincoln (NE): University of Nebraska; 2010 [cited 2015 Jul]. http://digitalcommons.unl.edu/bioscivaterfowlna/1

Technical Appendix Table. Summary of the number of disease outbreaks caused by highly pathogenic avian influenza virus, subtype H5, reported in North America according to species of wild bird

Family	Species	No. reports
Anseriformes	Mallard	16
Anseriformes	American wigeon	11
Anseriformes	Northern pintail	5
Anseriformes	Canada goose	5
Anseriformes	American green-winged teal	4
Anseriformes	Lesser snow goose	4
Anseriformes	Wood duck	3
Anseriformes	Northern shoveler	3
Falconiformes	Red-tailed hawk	2
Falconiformes	Cooper's hawk	2
Falconiformes	Bald eagle	1
Anseriformes	Ring-necked duck	1
Strigiformes	Snowy owl	1
Anseriformes	Gadwall	1
Anseriformes	Cinnamon teal	1
Falconiformes	Peregrine falcon	1

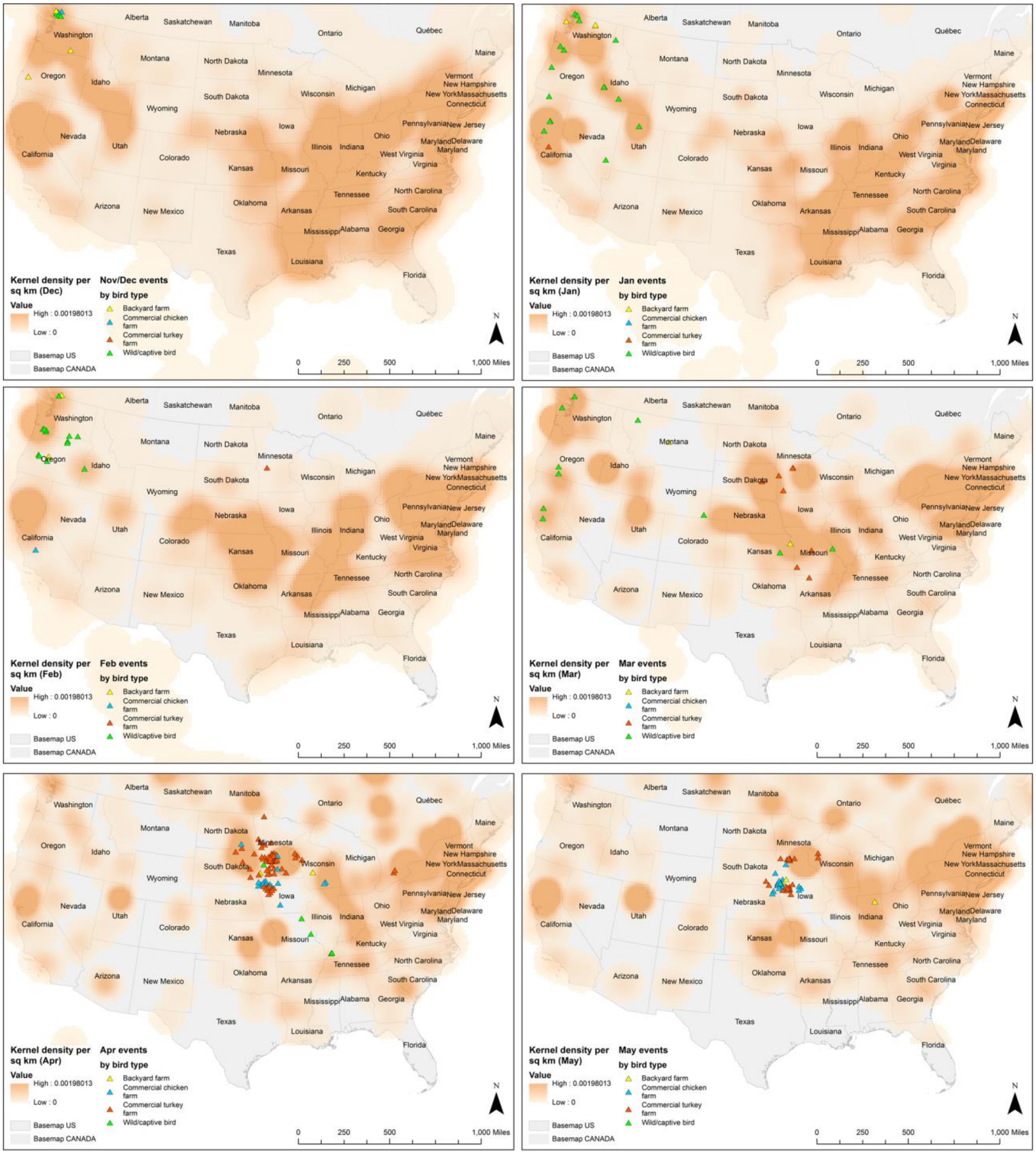

Technical Appendix Figure 1. Distribution of outbreaks caused by highly pathogenic avian influenza (HPAI) virus, subtype H5, layered over the monthly geographic distributions of reported wild bird encounters in December (A), January (B), February (C), March (D), April (E), and May (F). Yellow, blue, red, and green triangles represent HPAI H5 outbreak locations in backyard farms, commercial chicken farms, commercial turkey farms and wild/captive birds, respectively. Orange shading indicates the density of reported encounters with wild birds in December (A), January (B), February (C), March (D), April (E), and May (F). Darker shading indicates higher density of bird encounters.

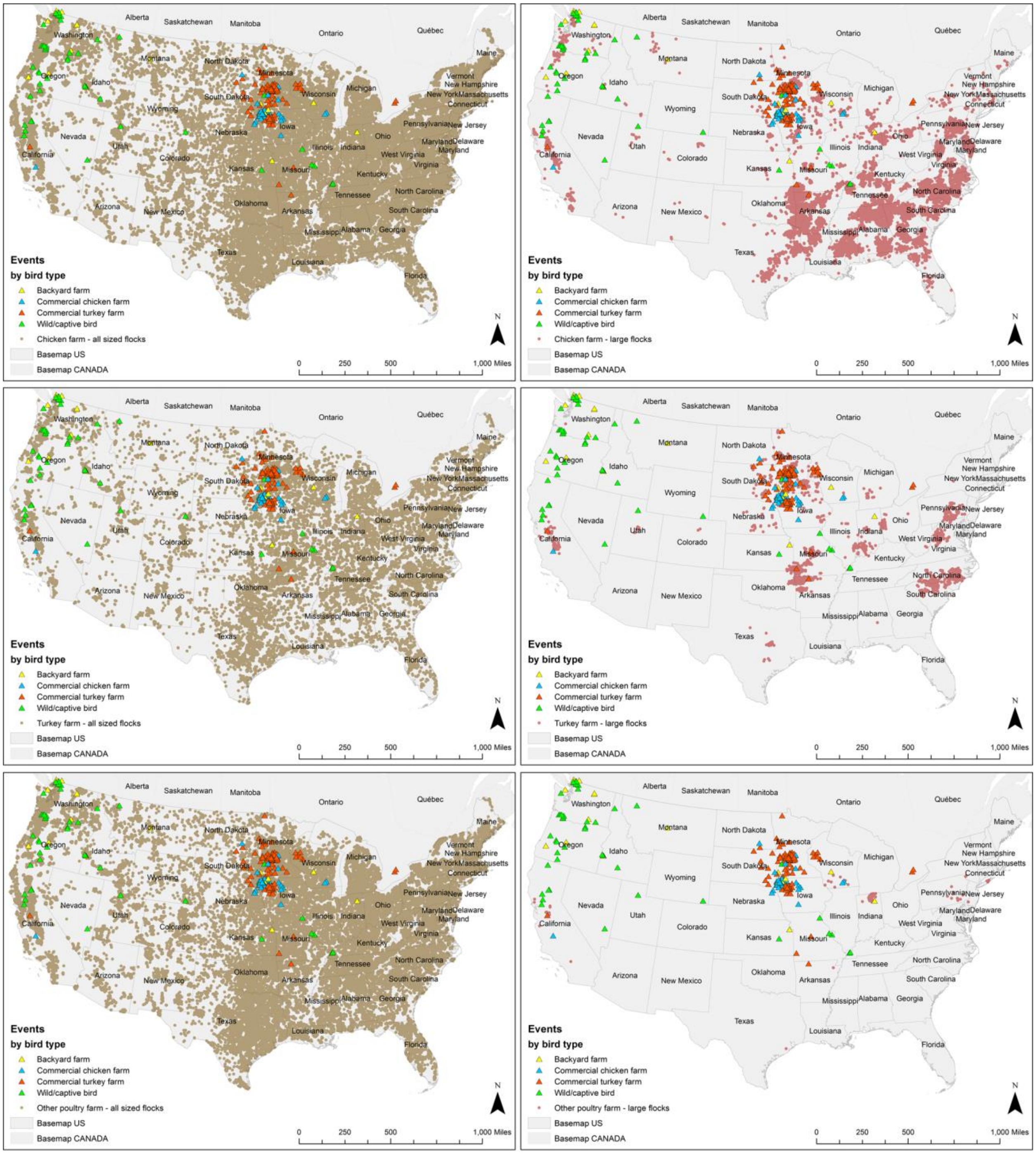

Technical Appendix Figure 2. Distribution of outbreaks caused by highly pathogenic avian influenza (HPAI) virus, subtype H5, layered over the geographic distributions of: all chicken farms (A), chicken farms with large flocks (B), all turkey farms (C), turkey farms with large flocks (D), all ducks/geese/other farms (E), and ducks/geese/other farms with large flocks (F). Yellow, blue, red, green triangles represent HPAI H5 outbreak locations in backyard farms, commercial chicken farms, commercial turkey farms, and wild/captive birds, respectively. Brown dots represent the estimated location of farms in the United States: all chicken farms (A), chicken farms with large flocks (B), all turkey farms (C), turkey farms with large flocks (D), all ducks/geese/other farms (E), and ducks/geese/other farms with large flocks (F).

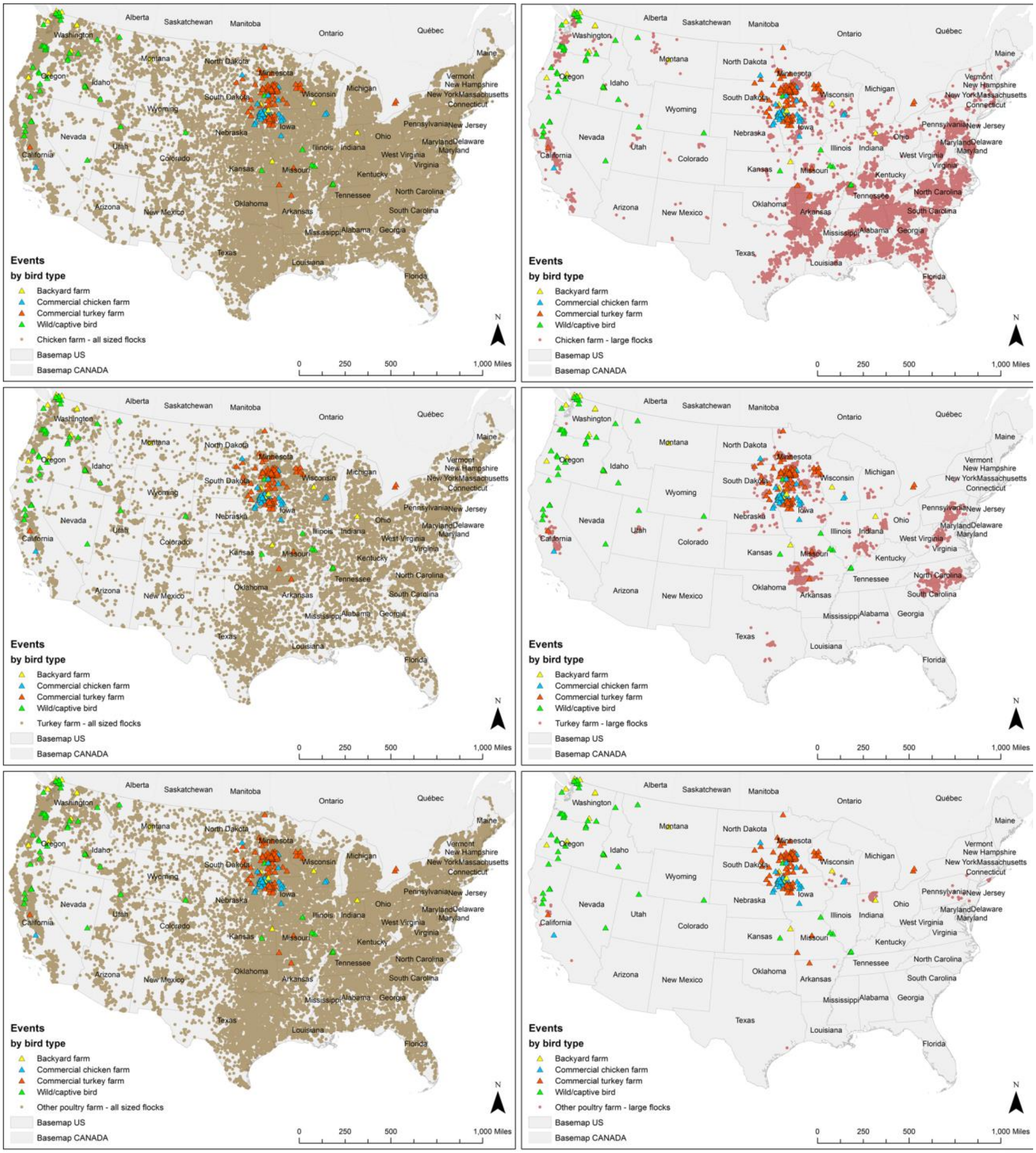

Technical Appendix Figure 3. Daily distribution of outbreaks caused by highly pathogenic avian influenza (HPAI) virus, subtype H5, from November, 30, 2014, through June 18, 2015. A) Distribution grouped by bird type: wild birds (n = 49), backyard farm birds (n = 20), commercial turkey farms (n = 154), and commercial chicken farms (n = 57) in North America. The 2 commercial mixed poultry farms were grouped as commercial chicken farms. B) Distribution by HPAI virus subtypes H5N2 (n = 256), H5N8 (n = 21), and H5N1 (n = 3).
